# The Clinical Values of Serum Markers in the Early Prediction of Hepatocellular Carcinoma

**DOI:** 10.1155/2017/5358615

**Published:** 2017-04-30

**Authors:** Bo Li, Boan Li, Tongsheng Guo, Zhiqiang Sun, Xiaohan Li, Xiaoxi Li, Han Wang, Weijiao Chen, Peng Chen, Yuanli Mao

**Affiliations:** ^1^Center for Clinical Laboratory, 302 Military Hospital, Beijing, China; ^2^Graduate Student Team, Medical University of PLA, Beijing, China

## Abstract

The early prediction values of diagnostic markers for hepatocellular carcinoma (HCC) are still unclear at present. This study evaluated the prediction value of ten serum markers in HCC. A total of 109 cases of hepatic cirrhosis patients were followed up for 36 months and the relationship between the lifetime risk of developing HCC and levels of serum markers was analyzed. 31.2 (34/109) percent of hepatic cirrhosis patients developed HCC during the study's timeframe. Higher alpha-fetoprotein (AFP), alpha-fetoprotein-L3 (AFP-L3), alanine aminotransferase (ALT), and AFP-L3/AFP ratio levels are potential risk factors for malignization in hepatic cirrhosis patients (RR = 2.99, 2.92, 2.72, and 2.34); serum Golgi protein 73 (GP73) level of hepatic cirrhosis patients decreased significantly after developing HCC (*t* = 2.212; *p* = 0.041). The detection of ALT, AFP, AFP-L3, and GP73 has a certain guiding significance to predict the risk of HCC in hepatic cirrhosis patients.

## 1. Introduction

Hepatocellular carcinoma (HCC) is the fifth leading cause of cancer death worldwide and about 500,000 people die of it each year [1]. More than 90% of HCC cases develop as a consequence of underlying liver diseases, and hepatic cirrhosis occurs in 80% of cases [2–4]. More than 60% of patients are diagnosed with late-stage disease after metastasis has occurred [5], resulting in an overall 5-year survival rate of <16% [6]. If appropriate treatments are performed in early stage, the 5-year survival rates of HCC patients will exceed 75% [7]. Thus, detection of HCC at an early stage significantly impacts outcomes in patients. The American Association for the Study of Liver Diseases (AASLD) once recommended that AFP and ultrasound examination were used for HCC surveillance in hepatic cirrhosis population, but analysis of recent studies shows that AFP determination lacks adequate sensitivity and specificity for effective surveillance [8]. Novel biomarkers are urgently needed for the screening of HCC to reduce its high mortality; many studies have reported that lens culinaris agglutinin reactive AFP (AFP-L3) and Golgi protein 73 (GP73) are effective for the HCC early diagnosis [9–11], but there has been a lack of clinical follow-up from hepatic cirrhosis stage to HCC. The goal of the present study is to estimate the risk prediction value of some serum markers during the progression from hepatic cirrhosis to HCC.

## 2. Materials and Methods

### 2.1. Subject

All study subjects were enrolled at 302 military hospital, Beijing, China, and were followed up during the study period of 36 months, until confirming HCC diagnosis or the date of study end (December 31, 2016). The study population included any hepatic cirrhosis patients over 30 years old who were identified as HBV or HCV infected patients for at least 5 years. Some patients with the following conditions were excluded: patients who were diagnosed with HCC at starting point of this study; patients with other systemic disease such as diabetes and hypertension; patients after surgery, interventional therapy, radiotherapy, chemotherapy, and other invasive treatment; patients suffering from severe complications such as upper gastrointestinal bleeding and hepatic encephalopathy. The final diagnosis was made by liver histopathology or MRI based on guidelines from Ministry of Health of the People's Republic of China [12] and guideline from the Chinese Society of Hepatology and the Chinese Society of Infectious Diseases [13, 14]. The study procedures were approved by the ethics committee of the 302 Military Hospital of China and written informed consent was obtained from each subject.

### 2.2. Laboratory Tests

A total of ten routine laboratory tests were chosen to be analyzed; they were albumin (ALB), total bilirubin (TBil), alanine transaminase (ALT), platelet count (PLT), prothrombin time (Pt(s)), prothrombin time activity (Pt(a)), AFP, GP73, AFP-L3, and AFP-L3/AFP ratio (L3/AFP). Clinical chemistry tests were applied by an automatic biochemical analyzer (AU5400, Olympus, Japan). PLT was detected using Hematology Analyzer (XE-1800, SYSMEX, Japan). Pt(s) and Pt(a) were measured in automated coagulation instrument (CA-7000, SYSMEX, Japan). AFP and AFP-L3 were measured by Automated Immunoassay Analyzer (COBAS6000, ROCHE, Switzerland). Kits for the enzyme-linked immunosorbent assay for GP73 were obtained from Hotgen Biotech (Beijing, China).

### 2.3. Study Protocol and Statistical Analysis

The incidences of HCC during the study period were analyzed by examination of medical records. Ten markers at starting point of this study were compared between patients with abnormal serum biomarkers levels, denoted as the positive groups, and those with normal levels, denoted as negative groups. The judgment criteria are as follows: ALB < 35 g/l, TBIL > 19 *μ*mol/L, ALT > 40 U/L, PLT < 100 × 10^9^/L, AFP > 10 ng/mL, PT(s) > 13 s, PT(a) < 75%, AFP-L3 > 1.0 ng/mL, GP73 > 150 ng/mL, and AFP-L3/AFP > 0.05. After 3 years' follow-up, cumulative incidence (CI) and relative risk (RR) of ten markers were calculated for each group to identify potential risk factors for HCC. A chi-square test was performed to compare the incidence rate between the positive and negative groups in our study cohort. All markers at starting point of this study were compared between patients who had developed HCC within 3 years and those who had not developed HCC to explore the early prediction value of serum markers. In order to investigate the dynamic change of the serum markers during the progression of HCC, we had compared all markers in HCC patients for two time points: starting point and the time they are diagnosed with HCC. Normally distributed data were analyzed with Student's *t*-tests. Other data were tested by the Wilcoxon method. To assess the role of all markers as diagnostic predictive markers for HCC, receiver operating characteristic curves (ROC) were plotted and the area under the curve (AUC) was calculated. All statistical analysis was performed using SPSS 14.0 software (SPSS, Inc., Chicago, IL).

## 3. Results

### 3.1. Study Sample

A total of 161 cases were diagnosed as hepatic cirrhosis during the study period. Fifty-two cases were excluded (35 were excluded due to history of other systemic diseases, 3 participants were excluded due to excessive missing data, and 14 patients with confirmed severe complications were excluded). Therefore, a total of 109 patients met the inclusion criteria and were analyzed. All participants had a mean age of 53.9 (SD = 9.7) years, were 60.6% male, 94.5% were with a history of HBV infection (see Table 1). During 36 months of follow-up, 34 out of 109 cirrhotic patients were confirmed to have HCC eventually (31.2%).

### 3.2. Early Warning Value of Serum Markers for HCC

**Figure 1:**
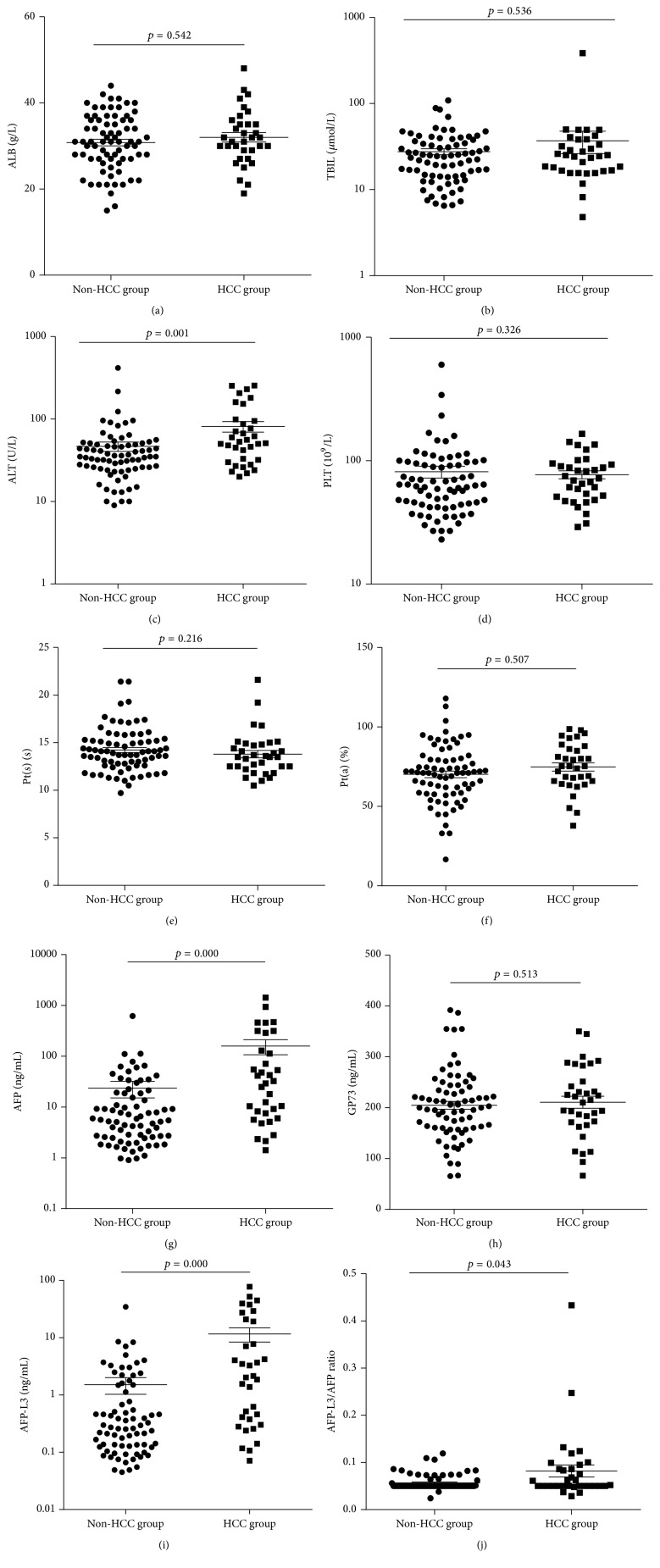
The difference of 10 biomarkers levels between patients who had developed HCC within 3 years and those who had not developed HCC.

We compared the serum markers levels at starting point between patients who had developed HCC and those who had not developed HCC; the results demonstrated that there were 4 markers, including AFP, AFP-L3, ALT, and AFP-L3/AFP ratio, that were statistically significant (*p* < 0.05); see Table 2 and Figure 1. It is evident that the increase of AFP, AFP-L3, and ALT levels in serum and AFP-L3/AFP ratio are potential precursors of HCC; thus regular monitoring of these markers seems necessary in hepatic cirrhosis patients.

The risk factors analysis showed that incidence rate of HCC in patients with high AFP, AFP-L3, ALT, and AFP-L3/AFP levels were extremely significantly higher than that those with normal levels (RR = 2.99, *p* = 0.000; RR = 2.92, *p* = 0.000; RR = 2.72, *p* = 0.001; RR = 2.34, *p* = 0.003). Our results revealed that cirrhotic patient with higher levels of AFP, AFP-L3, AFP-L3/AFP ratio, or ALT had a risk of developing HCC and these four markers were risk factors for HCC. In contrast to the previous studies, we find that high GP73 level seemed to be a protective factor for HCC, because elevated GP73 levels were associated with a lower risk of incident HCC (see Table 3).

### 3.3. Predictive Value of Serum Markers for HCC

**Figure 2:**
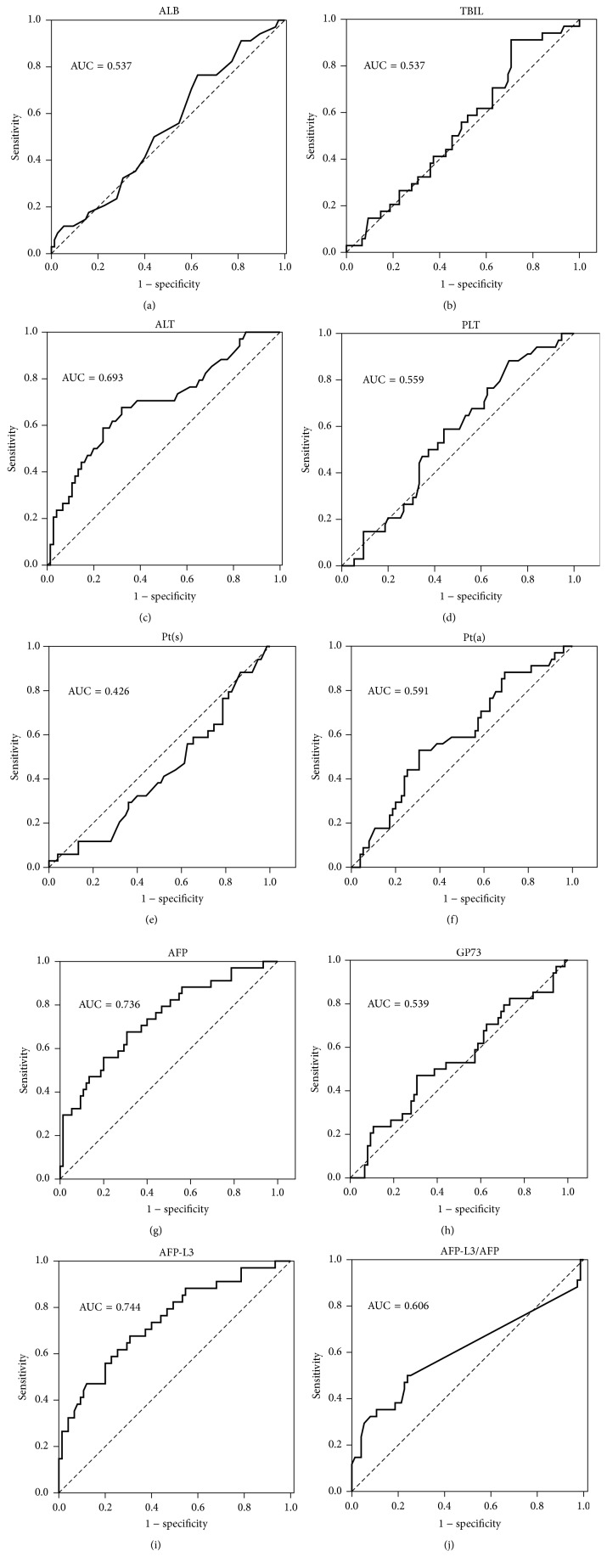
The ROC curves of 10 biomarkers for prediction of HCC in the cirrhotic patients.

**Figure 3:**
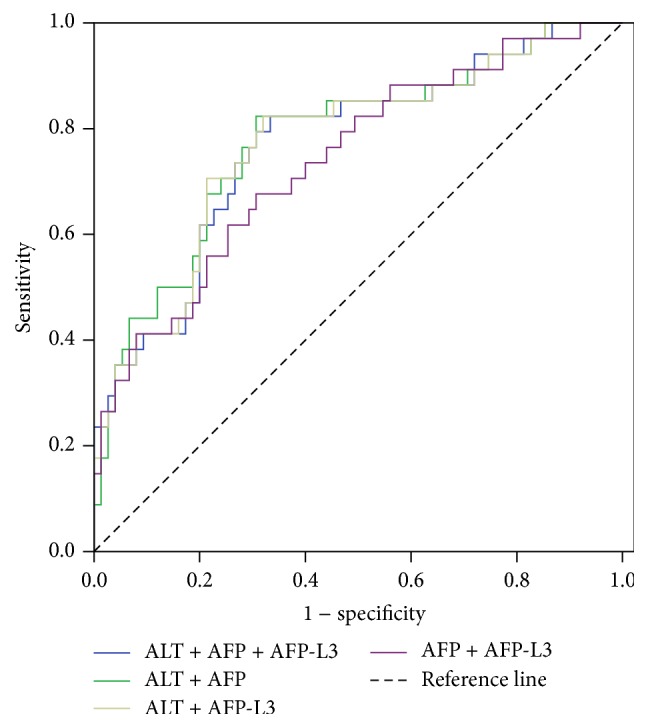
The multiple regression analysis with ALT, AFP, and AFP-L3 for predicting performance.

ROC analysis was used to determine whether serum markers are powerful to predict HCC in the cirrhotic population. The results showed that AFP, AFP-L3, and ALT had relatively good predictive power for HCC progression; AUC were 0.736, 0.744, and 0.693, respectively (see Table 4, Figure 2). The multiple regression analysis suggested that the combination of three markers could not significantly improve the predictive efficacy; the best combination was ALT and AFP, which obtained AUC of 0.780; see Table 4, Figure 3.

### 3.4. Dynamic Change of the Serum Markers during the Progression of HCC

**Figure 4:**
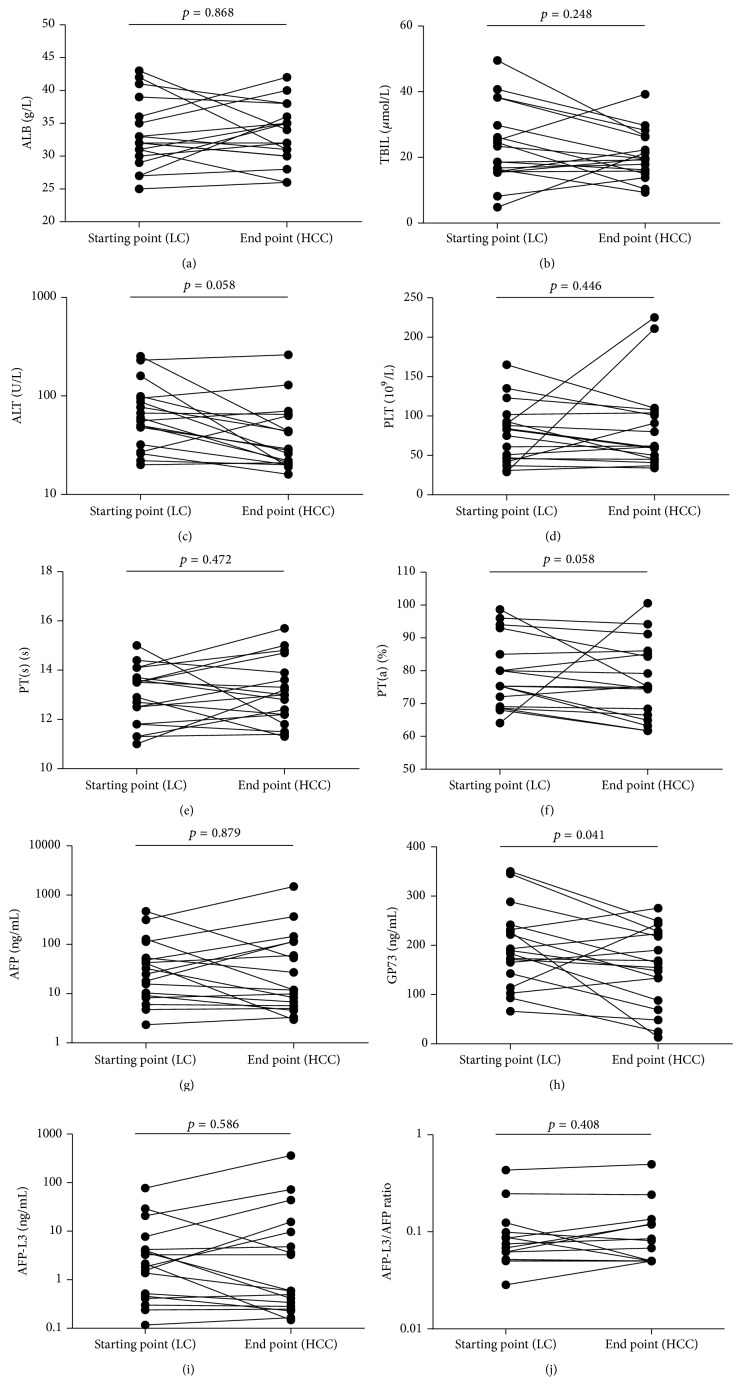
The plotting diagram of 10 biomarkers levels of patients who had developed HCC in starting point (hepatic cirrhosis) and end point (HCC).

Among the 34 HCC cases, 17 were excluded due to incomplete data or nonavailable data. We analyzed the dynamic change of the ten markers in other 17 cases during the progression of HCC, and we found that serum GP73 level was significantly decreased (*p* = 0.041) in patients when they were identified with HCC. The concentration of GP73 was 194.6 (66.12–350) ng/mL in hepatic cirrhosis and 154.2 (13.14–275.4) ng/mL in HCC. See Table 5 and Figure 4. It showed a gradually decreasing tendency of serum GP73 accompanied by the development of HCC from hepatic cirrhosis.

## 4. Discussion

In the past few decades, many promising candidate biomarkers for HCC had been found, but most of them were not applied to clinical diagnosis due to their limited practicability and high cost [15–19]. Nevertheless, these new markers have potential to be applied in clinical diagnosis for their higher sensitivity and specificity. So far, *α*-fetoprotein (AFP) and imaging technology (e.g., ultrasound or computed tomography) are two primary methods to diagnose HCC in hospitals. AFP has been used as a serum marker for HCC for many years, but its sensitivity was only about 39%–65% [20]. AFP-L3, which is the main glycoform of AFP in the serum of HCC, was proven to be an excellent biomarker with sensitivity 75% to 96.90%. High percentage of AFP-L3 has been shown to be associated with poor differentiation and biologically malignant characteristics, worse liver function, and larger tumor mass; some experts thought that the AFP-L3/AFP ratio is more helpful in diagnosis and prognosis of HCC [21, 22]. However, Miura and his coworkers showed that AFP-L3 could not provide an entirely satisfactory solution to detect HCC at the early stage [23]. Our study showed that patients with higher levels of AFP, AFP-L3, AFP-L3/AFP ratio, and ALT have higher risk of developing HCC than those with normal levels of these markers, suggesting that these four markers are potential precursors of HCC in hepatic cirrhosis patients and that serum AFP, AFP-L3, AFP-L3/AFP ratio, and ALT may be useful markers for indicating the development of HCC.

GP73 is a resident Golgi-specific membrane protein expressed by biliary epithelial cells in normal liver. A meta-analysis reported that GP73 is a valuable serum marker that seems to be superior to AFP and can be useful in the diagnosis and screening of HCC [11]. However, Tian et al. [24] indicated that GP73 elevated not only in HCC but also in other chronic liver diseases such as hepatic cirrhosis and hepatitis; even more, the concentration of GP73 in HCC (median = 107.3 *μ*g/L) was significantly lower than hepatic cirrhosis (median = 141.2 *μ*g/L) patients, but their conclusions may suffer from sample selection biases. In our study, followed-up experiments were conducted to assess the dynamic change of the serum markers during the progression of HCC. Our findings further confirm that serum GP73 level was significantly decreased during the progression of HCC. Some research results found that GP73 protein and mRNA expression increase gradually in chronic liver diseases, not only in the hepatocytes, but also in activated stellate cells which are the most important cell type in hepatic cirrhosis [25–27]. Therefore, maximal GP73 concentrations were observed in hepatic cirrhosis rather than in HCC.

## 5. Conclusion

Although this study is limited by the small sample size and short study duration, our data suggest that higher serum levels of AFP, AFP-L3, AFP-L3/AFP ratio, and ALT were risk factors associated with the development of HCC and the detection of GP73 has a certain guiding significance to predict the risk of HCC in hepatic cirrhosis patients; regular monitoring of these serum markers in hepatic cirrhosis patients is necessary.

## Figures and Tables

**Table 1:** The demographic data of the patients.

	HCC-group	Non-HCC group	*p* value
Gender			
Male	22	44	0.550
Female	12	31	
Age (years)	56 (35–83)	52 (37–74)	0.118

**Table 2:** The statistical analysis of the levels of serum markers between HCC group and non-HCC group.

Markers	HCC group		Non-HCC group		*T*/*Z* values	*p* values
	*n*	Statistical description	*n*	Statistical description		
ALB (g/L)	34	32.0 ± 6.32	75	30.8 ± 6.5	0.897	0.542
TBIL (*μ*mol/L)	34	24.85 (4.8–385.6)	75	24.1 (6.5–109)	−0.618	0.536
ALT (IU/L)	34	52 (20–254)	75	35 (9–416)	−3.212	0.001
PLT (10^9^/L)	34	70.5 (29–165)	75	62 (23–599)	−0.981	0.326
Pt(s) (s)	34	13.5 (10.5–21.6)	75	14.00 (9.7–21.4)	−1.237	0.216
Pt(a) (%)	34	74.8 ± 15.0	75	70.2 ± 18.0	1.313	0.507
AFP (ng/mL)	34	30.88 (1.41–1432)	75	5.62 (0.9–617.4)	−3.944	0.000
GP73 (ng/mL)	34	212.6 (66.12–350)	75	202.8 (65.38–391.9)	−0.654	0.513
AFP-L3 (ng/mL)	34	2.09 (0.07–77.45)	75	0.273 (0.05–34.4)	−4.068	0.000
AFP-L3/AFP ratio	34	0.051 (0.03–0.43)	75	0.05 (0.02–0.12)	−2.019	0.043

Normally distributed data were reported as mean ± SD. Other data were reported as median (minimum, maximum); *n*: number of samples.

**Table 3:** Analysis of risk factors for the development of HCC based on the levels of serum tumor markers.

Markers		HCC (*n*)	Non-HCC (*n*)	Cumulative incidence (CI)	Relative Risk (RR)	Attributable Risk (AR)	Attributable Risk Percent (ARP)	Chi-Square	*p* Value
ALB (g/l)	Positive groups	23	54	29.87%	1.06	1.75%	5.84%	0.033	0.855
	Negative groups	9	23	28.13%					

TBIL (*μ*mol/L)	Positive groups	21	44	32.31%	1.09	2.76%	8.55%	0.093	0.760
	Negative groups	13	31	29.55%					

ALT (U/L)	Positive groups	24	30	44.44%	2.72	28.08%	63.18%	10.178	0.001
	Negative groups	9	46	16.36%					

PLT (10^9^/L)	Positive groups	27	59	31.40%	1.20	5.31%	16.91%	0.242	0.623
	Negative groups	6	17	26.09%					

PT(s) (s)	Positive groups	20	49	28.99%	0.97	−1.01%	−3.50%	0.013	0.911
	Negative groups	12	28	30.00%					

PT(a) (%)	Positive groups	17	52	28.81%	0.77	−8.69%	−30.15%	2.020	0.155
	Negative groups	15	25	37.50%					

AFP (ng/mL)	Positive groups	24	25	48.98%	2.99	32.59%	66.53%	13.511	0.000
	Negative groups	10	51	16.39%					

GP73 (ng/mL)	Positive groups	27	64	29.67%	0.89	−3.66%	−12.35%	0.096	0.757
	Negative groups	6	12	33.33%					

AFP-L3 (ng/mL)	Positive groups	22	20	52.38%	2.92	34.47%	65.81%	14.292	0.000
	Negative groups	12	55	17.91%					

L3/AFP ratio	Positive groups	18	19	48.65%	2.34	27.82%	57.18%	8.958	0.003
	Negative groups	15	57	20.83%					

HCC: patients who had developed HCC during the study period; non-HCC: patients who had not developed HCC during the study period; *n*: number of patients.

**Table 4:** The predictive value of all markers and combination of 3 markers for HCC in cirrhotic patients.

Markers	Area under ROC curve	Standard error	*p* value	95% confidence interval	Cut-off	Sensitivity	Specificity
ALB (g/l)	0.537	0.059	0.534	0.423–0.652	28.5	0.765	0.373
TBIL (*μ*mol/L)	0.537	0.058	0.537	0.423–0.651	15.15	0.912	0.293
ALT (U/L)	0.693	0.056	0.001	0.582–0.803	44.5	0.676	0.680
PLT (10^9^/L)	0.559	0.057	0.327	0.447–0.670	45.5	0.882	0.720
PT(s) (s)	0.426	0.058	0.216	0.311–0.540	21.5	0.029	1.000
PT(a) (%)	0.591	0.058	0.128	0.477–0.705	75	0.529	0.693
AFP (ng/mL)	0.736	0.052	0.000	0.634–0.839	10.28	0.676	0.693
GP73 (ng/mL)	0.539	0.061	0.513	0.419–0.659	221.85	0.471	0.693
AFP-L3 (ng/mL)	0.744	0.052	0.000	0.642–0.846	0.514	0.676	0.693
L3/AFP ratio	0.606	0.065	0.077	0.478–0.734	0.052	0.500	0.760
ALT + AFP + AFP-L3	0.770	0.050	0.000	0.672–0.868	0.23	0.824	0.667
ALT + AFP	0.780	0.049	0.000	0.683–0.877	0.24	0.824	0.693
ALT + AFP-L3	0.773	0.050	0.000	0.676–0.871	0.23	0.824	0.680
AFP + AFP-L3	0.740	0.052	0.000	0.638–0.842	0.23	0.676	0.693

**Table 5:** The dynamic change of serum markers concentrations before and after HCC occurred.

Markers	Starting point (hepatic cirrhosis)		End point (HCC)		*T*/*Z* values	*p* values
	*n*	Statistical description	*n*	Statistical description		
ALB (g/l)	17	32 (25–43)	17	33 (26–42)	−0.166	0.868
TBIL (*μ*mol/L)	17	21 (4.8–49.5)	17	19.6 (9.3–39.2)	−1.154	0.248
ALT (U/L)	17	58 (20–253)	17	28.5 (16–262)	−1.895	0.058
PLT (10^9^/L)	17	79 (29–165)	17	61.5 (34–225)	−0.762	0.446
PT(s) (s)	17	13.2 (11–15)	17	13 (11.3–15.7)	−0.719	0.472
PT(a) (%)	17	75.3 (64.1–98.7)	17	75.1 (61.7–100.6)	−1.894	0.058
AFP (ng/mL)	17	74.6 (2.34–469.9)	17	136.2 (2.96–1501)	−0.152	0.879
GP73 (ng/mL)	17	194.6 (66.12–350)	17	154.2 (13.14–275.4)	2.212	0.041
AFP-L3 (ng/mL)	17	8.8 (0.12–77.45)	17	28.8 (0.15–361.5)	−0.544	0.586
AFP-L3/AFP ratio	17	0.09 (0.03–0.43)	17	0.10 (0.05–0.50)	−0.848	0.408

Starting point: start time of this study; end point: the time the patients were diagnosed HCC; *n*: number of patients.
